# Supplementary material for: Efficient preservation of sprouting vegetables under simulated microgravity conditions
Source: PLoS One. 2020 Oct 15;15(10):e0240809. doi: 10.1371/journal.pone.0240809 (PMC7561153; doi:10.1371/journal.pone.0240809)
Supplement: Attachment — Submitted filename: Response to Reviewers.docx [file pone.0240809.s001.docx]

October 2^nd^, 2020

Dear Dr. Sakamuri V. Reddy,
Academic Editor,
PLOS ONE

Dear Editor,

Thank you for giving us the opportunity to re-submit our manuscript entitled “**Efficient preservation of sprouting vegetables under simulated microgravity conditions**” for publication in *PLOS ONE.* We found your and the reviewer’s comments very helpful, and have provided our responses to each of these below.

We believe that we have adequately addressed the concerns raised in our revisions of this manuscript However, if you consider these to be insufficient, please indicate where further revision is required and we will gladly accommodate your requests.

Sincerely,

Yoshio Makino

Graduate School of Agricultural and Life Sciences, The University of Tokyo

Yayoi 1-1-1, Bunkyo-ku, Tokyo, Japan, 113-8657

Fax: +81-3-5841-8174

Email: amakino@mail.ecc.u-tokyo.ac.jp

**Responses to Editor**

In response to “Additional Editor Comments”, we have made the following changes to the manuscript.

Comment

The authors demonstrate that simulated microgravity is effective over conventional methods such as refrigeration to preserve the nutritional value of sprout vegetables. They should consider rephrasing the title, ex., “Efficient preservation of sprouting vegetables under simulated microgravity conditions”.

Response

The original title “Depression of mass loss … under simulated microgravity” has been revised as “Efficient preservation of sprouting … simulated microgravity conditions” in the revised manuscript with track changes.

Comment

They have observed that vegetable sprouts placed in a simulated microgravity environment preserve the mass maintaining freshness over time. However, they should clarify the implications of the study with respect to space environment or during long-term space flight missions in conclusions of Abstract, Introduction and Conclusions noted (p.11; line 262).

Response

The sentence “This founding will support long-term … shelf life of sprout vegetables.” has been inserted in line 33-34 in “Abstract” section. The sentences “Since a stable supply of food is … by extending the shelf life of the crop.” has been inserted in line 86-89 in “Introduction” section. The sentence “This founding will support long-term … shelf life of sprout vegetables.” has been inserted in line 292-293 in “Conclusion” section.

Comment

Methods-(p.6; line 136) they should clarify the section for statistical significance considered.

Response

Statistical significance levels as “*p* < 0.05” for Student’s t-test and two-way analysis of variance were inserted in lines 143 and 144 in the revised manuscript with track changes.

Comment

Also, they should provide microgravity simulation method with citation. Provide a rationale for Fig.2 and Fig.4 under Results and Discussion (p.6; line 142 & p.7; line 171).

Response

Microgravity simulation method and rational have been provided as “The principle of operation of the microgravity device is … to 0 m s^−2^ (Uemura and Yuge, 2011).” in lines 152-153, and “The principle of operation of the microgravity device is … to 0 m s^−2^ (Uemura and Yuge, 2011). Normal gravity was 9.8 m s^−2^ (gravitational acceleration on earth).” In lines 179-181 in the revised manuscript with track changes with citation. In this research, the word “simulated” is equivalent to “quasi”.

Comment

Fig.3 provided appears to be an illustration. They should show real picture or image of mung bean sprouts under experimental conditions.

Response

Fig. 3 has been revised by providing real images. Graphical abstract has also been revised by providing real images as the same as revised Fig. 3.

Comment

They should explain the Fig.5B results clearly.

The figure shows closed bars and open bars representing normal and microgravity conditions, however no significant change in Soybean. They should explain the results and Figure.5 clearly for the decrease in mass loss under microgravity conditions.

Response

The sentence “This mechanism of mass loss may not be affected dependent of gravity levels.” has been inserted in lines 240-241 in the revised manuscript with track changes to clearly explain the reason why no significant change in soybean was observed.

The sentence “According to the results in Fig. 4, … under the microgravity condition. However,” has been inserted in lines 235-236 in the revised manuscript with track changes to clearly explain the decrease in mass loss under microgravity conditions.

Comment

I suggest the authors to proofread and take language assistance in preparing the manuscript.

Response

A native speaker belonging to “Enago, Crimson Interactive Pvt. Ltd” has supported us by correcting English language as the attached certificate.

**Responses to Reviewer #1**

In response to the editor’s suggestions”, we have made the following changes to the manuscript.

Comment

This is a clearly written, easy to read research article. It approaches the concept of using a clinostat from a completely new angle - to increase shelf life and improve food preservation of sprouts.

Response

We express our sincere thanks for your great evaluation to our research.

Comment

While not necessary to add to this current work, I noted the mention of ethylene scrubbing as a means of food preservation, and I am curious if the author has considered the problems that plants often encounter with ethylene in an enclosed environment, specifically on clinostats and in the true microgravity of spaceflight.

Response

In the present study, the samples were stored under the atmosphere without ethylene because the inner space is connected to outer space through the vent hole (Fig. 1(A)). It may be important that vegetables are stored under the atmosphere without ethylene by using vent hole as the same as this study or the scrubber in the true microgravity of spaceflight.

Comment

Ray Wheeler has several publications on this topic, and it seems odd that they were not addressed nor mentioned. I encourage them to look into the topic.

Response

Thanks for telling us that Dr Wheeler has made a lot of research on plant biology under microgravity. In particular, a review article (Paul, Wheeler et al., Am. J. Botany, 100, 226-234, 2013) introduced many useful research results in the past and provided various new information. I quoted the review article as “Since a stable supply of food … in recent years (Paul et al., 2013).” in lines 86-88, and “Paul et al. (2013) introduced many reports … and altered chlorophyll a/b ratios.” in lines 272-274 in the revised manuscript with track changes.
